# BeEM: fast and faithful conversion of mmCIF format structure files to PDB format

**DOI:** 10.1186/s12859-023-05388-9

**Published:** 2023-06-20

**Authors:** Chengxin Zhang

**Affiliations:** Department of Computational Medicine and Bioinformatics, University of Michigan, Ann Arbor, MI 48109 USA

**Keywords:** Protein structure, PDB format, mmCIF format

## Abstract

**Background:**

Although mmCIF is the current official format for deposition of protein and nucleic acid structures to the protein data bank (PDB) database, the legacy PDB format is still the primary supported format for many structural bioinformatics tools. Therefore, reliable software to convert mmCIF structure files to PDB files is needed. Unfortunately, existing conversion programs fail to correctly convert many mmCIF files, especially those with many atoms and/or long chain identifies.

**Results:**

This study proposed BeEM, which converts any mmCIF format structure files to PDB format. BeEM conversion faithfully retains all atomic and chain information, including chain IDs with more than 2 characters, which are not supported by any existing mmCIF to PDB converters. The conversion speed of BeEM is at least ten times faster than existing converters such as MAXIT and Phenix. Part of the reason for the speed improvement is the avoidance of conversion between numerical values and text strings.

**Conclusion:**

BeEM is a fast and accurate tool for mmCIF-to-PDB format conversion, which is a common procedure in structural biology. The source code is available under the BSD licence at https://github.com/kad-ecoli/BeEM/.

## Background

The macromolecular Crystallographic Information File (mmCIF, also known as PDBx/mmCIF) format [1] was introduced to the PDB database as its new standard for structure data deposition. The reason for the replacement of the previous official format (the legacy PDB format) by mmCIF is that all data fields in a PDB format file have fixed width, e.g., 5 characters and 1 character for an atom number and a chain identifier (chain ID), respectively. This limits the maximum number of atoms and chains in a PDB file to 99,999 and 62, respectively. By contrast, the mmCIF format represents structure information as a space-separated tabular text file, where each data field can have unlimited length. This enables an mmCIF file to represent highly complicated structures with more atoms and chains than a PDB file. As of October 2022, for example, there are 3254 structures in the PDB database that are available as mmCIF but not as standard PDB format files.

Despite the advantages of mmCIF, for legacy reasons, the PDB format is still the only supported format for many bioinformatics applications ranging from side-chain packing [2, 3] and tertiary structure prediction [4] to structure alignment [5, 6] and function prediction [7, 8]. Even for some programs that support both mmCIF and PDB formats, PDB is still the preferred format due to smaller input size and faster file reading speed thanks to its fixed-width nature. For example, alignment of mmCIF structure by the TM-align program [9] is twice as slow as aligning PDB structures.

To fulfill the need to use these programs on structures that are not available as a single PDB file, the PDB database provides “Best Effort/Minimal” PDB format, which splits a large mmCIF files into multiple smaller PDB files, each with up to 99,999 atoms and up to 62 chains. A mapping file is also provided to map each original chain ID with two or more characters to a single-character chain ID in the split PDB file. The split PDB files and the mapping files are then bundled into a single TAR file. Despite its ability to encode arbitrarily large structures, there is not yet a publicly available webserver or standalone program for the generation of Best Effort/Minimal PDB files. Moreover, for structures without standard PDB format, Best Effort/Minimal files are not always available from the PDB database, such as PDB ID: 7nwg, 7nwh, and 7nwi [10].

**Fig. 1:**
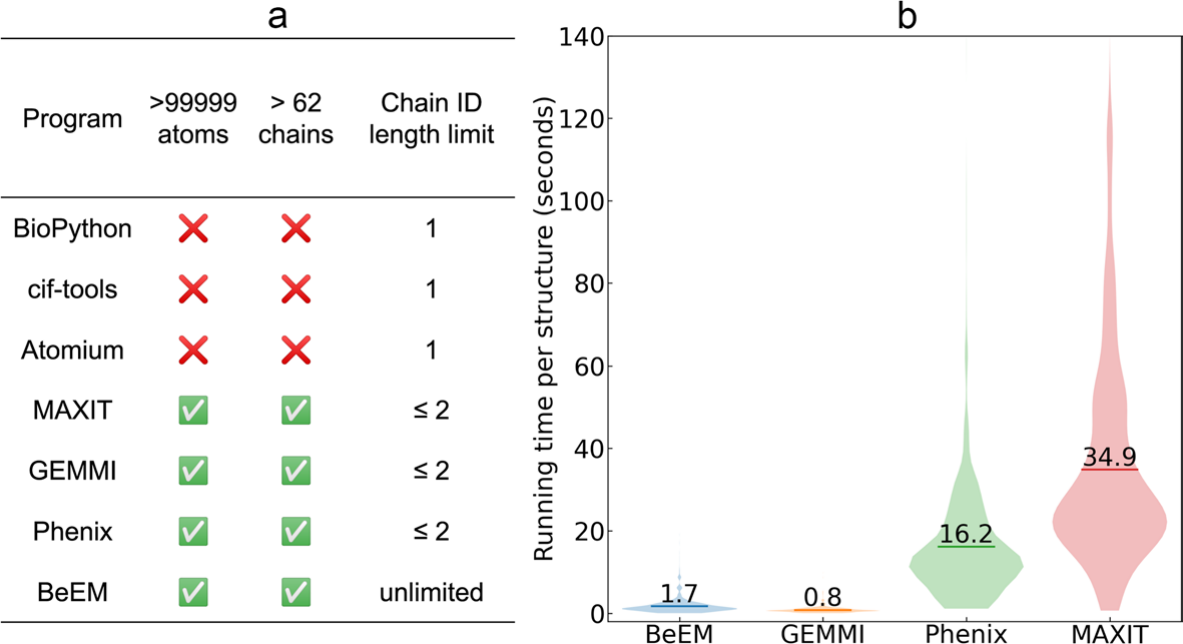
Comparison between BeEM and existing methods. a. Limitations on the number of atoms and chains by different mmCIF to PDB conversion programs. Here, “Phenix” stands for the phenix.cif_as_pdb program from the Phenix package. b. Running time of BeEM and three third-party programs for mmCIF to PDB format conversion. Horizontal bars indicate the average running time. BioPython, cif-tools and Atomium are not included because they cannot correctly generate PDB file for any of the input mmCIF structures

To this end, several converters from mmCIF to PDB have been developed by the community (Fig. 1a). Among these conversion programs, BioPython [11], cif-tools (https://github.com/PDB-REDO/cif-tools) and Atomium [12] can only handle up to one character in chain ID, again limiting the number of distinct chains in the output PDB file to 62. MAXIT (https://sw-tools.rcsb.org/apps/MAXIT), GEMMI [13] and Phenix [14], on the other hand, handle two-character chain IDs in the output PDB files by occupying the usually unused column 21 in addition to column 22, the latter of which is reserved for the chain ID. MAXIT, GEMMI and Phenix are, however, still unable to handle the 1036 structures from the PDB with chain IDs exceeding two characters.

To address these issues, this study proposed the Best Effort/Minimal (BeEM) program to convert mmCIF structure files to PDB files. It is currently the only open-source implementation for generation of Best Effort/Minimal PDB bundle files.

## Implementation

BeEM is written in C++ without external dependencies. Following the Best Effort/Minimal file specification (https://www.rcsb.org/docs/general-help/structures-without-legacy-pdb-format-files), BeEM reads the _struct_keywords, _audit_author, _citation_author/_citation, _cell/_symmetry, _atom_sites, and _atom_site_anisotrop records from the input mmCIF files and outputs the HEADER, AUTHOR, JRNL, CRYST1, SCALE/ATOM/HETATM and ANISOU records in the PDB format files, respectively. Optionally, it can read the _entity_poly/_entity_poly_seq and _struct_ref/_struct_ref_seq records of the mmCIF files and convert them to SEQRES and DBREF records in PDB format, respectively. To improve the speed, whenever possible, numerical values in the mmCIF files such as residue number, atomic coordinates, B-factors and occupancies are read as strings and padded to fixed width strings, without converting to integers or float numbers before reformatted to text for output as in previously developed tools [11–14].

If the mmCIF input contains chain IDs with two or more characters, the user can choose to either output Best Effort/Minimal PDB files that map multi-character chain IDs to single character IDs, or output a Phenix-style PDB file that retains two-character chain IDs. Since BeEM output does not contain the SHEET record for beta sheet, it is not limited by complex beta sheet topology (e.g., PDB 4dcb chain A). For chains with > 99,999 atoms (e.g., PDB 4v5x chain AA), users can choose to either split a single chain into two or more files or output a single file for the long chain with duplicated atom numbers. Since PDB format file cannot assign a unique atom index to every atom if the structure contains > 99,999 atoms, BeEM does not parse covalent bond information (e.g., SSBOND and CONECT records in the PDB file), which requires unique indexes for the bound atoms.

BeEM is designed to be future proof. For example, although the PDB database announced the plan to expand residue names of some new ligands into 5 characters (https://www.rcsb.org/news/630fee4cebdf34532a949c34), residue names in all currently available structures in the PDB database have up to 3 characters. Nonetheless, BeEM is designed to map residues names with > 3 characters to a set of reserved chemical component IDs (01–99, DRG, INH, LIG) that will never be used in the PDB database, so that the coordinates of ligands with long residue names can still be represented. Similarly, although the longest chain in the current PDB database has only 7249 residues (PDB 4v5x chain AA), BeEM can technically handle very large chains with up to 99,999 residues. In this case, the first 4 digits of a 5-digit residue number will occupy column 23–26 in the PDB file corresponding to the usual location for the residue number, while the last digit will occupy column 27 usually used for insertion code.

## Results

BeEM, together with MAXIT, GEMMI, and Phenix, are benchmarked on a large dataset of 2218 structures from the PDB database that are available as mmCIF and Best Effort/Minimal files but not PDB format files. Although BeEM can handle any mmCIF format input, MAXIT, GEMMI and Phenix only handles up to two characters in the chain IDs. Therefore, only structures with up to two characters in their chain IDs are included in this dataset. On average, BeEM takes 1.7 s to convert an mmCIF file, which is slower than GEMMI but 9.6 and 20.7 times faster than Phenix and MAXIT, respectively (Fig. 1b). Part of the reason for the faster speed of BeEM compared to existing program is that it avoids conversion of numerical values encoded by the input text file (e.g., atomic coordinates) to and from float numbers during conversion. The Best Effort/Minimal files from BeEM are compatible with popular structure analysis tools (Table 1) [9, 15–18], the majority of which are not yet compatible with mmCIF format [3, 5, 7, 19–23], including several programs that are developed or updated very recently [2, 6, 8]. Additionally, BeEM was tested on all 203,607 mmCIF format structures from the PDB database to confirm that correct results can be generated for diverse mmCIF files.

**Table 1:** Compatibility between popular structural bioinformatics program and different structure file format

Task	Program	Citation	File format compatibility		
			mmCIF	PDB	Best effort/minimal PDB
Structure alignment	DALI	[6]	No	Yes	Yes
	CE	[5]	No	Yes	Yes
	MICAN	[19]	No	Yes	Yes
	SSM	[15]	Yes	Yes	Yes
	TM-align	[9]	Yes*	Yes	Yes
	US-align	[16]	Yes	Yes	Yes
Structure-based function prediction	COFACTOR	[7]	No	Yes	Yes
	COACH-D	[20]	No	Yes	Yes
	ProFunc	[21]	No	Yes	Yes
	DeepFRI	[8]	No	Yes	Yes
Secondary structure assignment	DSSP	[17]	Yes	Yes	Yes
	STRIDE	[22]	No	Yes	Yes
Full atomic structure reconstruction	PULCHRA	[23]	No	Yes	Yes
	PDBFixer	[18]	Yes	Yes	Yes
	FASPR	[2]	No	Yes	Yes
	SCWRL	[3]	No	Yes	Yes

*Although TM-align is compatible with mmCIF format input, it runs approximately twice slower when using mmCIF than using PDB format input

## Conclusions

Despite advocacy of the new mmCIF format by the PDB database, the legacy PDB format remains the preferred format for many bioinformatics pipelines due to either historical reasons or performance considerations. This discrepancy necessitates the frequent conversion between mmCIF and PDB format files. To this end, the BeEM program was developed, which is comparable to or much faster than existing conversion programs in terms of speed, partly thanks to its unique numerical value parsing approach. BeEM can parse complicated structures with long chain IDs and expanded residue names that cannot be otherwise handle by existing methods. It is also the first publicly available program that is fully compliant with the Best Effort/Minimal file format specification. These advantages make BeEM a particular useful tool for structural bioinformatics.

## Data Availability

The dataset and source code to benchmark BeEM in this study are available at https://doi.org/10.5281/zenodo.7215696. Project name: BeEM. Project home page: https://github.com/kad-ecoli/BeEM/. Operating system(s): Linux, MacOS and Windows. Programming language: C++ . Other requirements: Apart from the standard C++ 98 libraries, BeEM does not dependent on any external library. While BeEM natively read uncompressed mmCIF files, it uses the “gunzip” program when reading gzip-compressed mmCIF files. License: BSD 2-clause. Any restrictions to use by non-academics: No restrictions.

## Abbreviations

PDB: Protein data bank
mmCIF: Macromolecular crystallographic information file
POSIX: Portable operating system interface
WSL: Windows subsystem for Linux
